# Pesticide Testing on Human Subjects: Weighing Benefits and Risks

**DOI:** 10.1289/ehp.7720

**Published:** 2005-03-16

**Authors:** David B. Resnik, Christopher Portier

**Affiliations:** ^1^Office of the Scientific Director, and; ^2^National Toxicology Program, National Institute of Environmental Health Sciences, National Institutes of Health, Department of Health and Human Services, Research Triangle Park, North Carolina, USA

**Keywords:** Environmental Protection Agency, ethics, Food Quality Protection Act, human subjects research, pesticide testing

## Abstract

In the debate surrounding testing pesticides on human subjects, two distinct positions have emerged. The first position holds that pesticide experiments on human subjects should be allowed, but only under stringent scientific and ethical standards. The second position asserts that these experiments should never be allowed. In this article, we evaluate what we consider to be the strongest argument for the second position—namely, that the benefits of the experiments are not significant enough to justify the risks posed to healthy subjects. We challenge this argument by examining the benefits and risks of testing pesticides on human subjects. We argue that a study that intentionally exposes humans subjects to pesticides should be permitted if *a*) the knowledge gained from the study is expected to promote human health; *b*) the knowledge cannot be reasonably obtained by other means; *c*) the study is not expected to cause serious or irreversible harm to the subjects; and *d*) appropriate safeguards are in place to minimize harm to the subjects.

## Background

Although private companies have tested pesticides on human subjects since the 1960s, the public debate about the ethics of such experiments began to simmer in 1998, when the Environmental Working Group (EWG) released a report titled *The English Patients: Human Experiments and Pesticide Policy* 1998. According to the report, the companies exposed volunteers to various insecticides to determine safety levels for exposure to these compounds. One of the experiments mentioned in the report involved the oral administration of dichlorvos to 53 subjects. Another experiment administered orange juice laced with aldicard to 47 subjects (EWG 1998). The media soon reported other pesticide experiments conducted elsewhere. In one experiment conducted by Novartis, managers for the company ingested diazinon. Experiments conducted by Novartis and Dow AgroSciences each used 60 paid volunteers (Gorovitz and Robertson 2000). In a study sponsored by Dow AgroSciences, dozens of college-age volunteers were paid $460 to swallow a pill containing chlorpyrifos, a roach poison (Shogren 2001).

The EWG report recommended that the U.S. Environmental Protection Agency (EPA) conduct a comprehensive review of its human research policies and issue a moratorium on the acceptance of data derived from privately funded (or third party) human experiments. In 2000, the U.S. EPA announced that it would not accept any pesticide data derived from privately funded toxicology research on human subjects until the ethical and regulatory issues were resolved (Lockwood 2004). In 2001, the U.S. EPA asked the National Research Council (NRC) to examine these issues; the U.S. EPA issued an Advance Notice of Proposed Rule-making in May 2003, before the NRC had completed its report (U.S. EPA 2003). In the notice, the U.S. EPA requested public comments on many different issues concerning industry-funded human studies submitted to the agency. The agency did not unconditionally endorse applicability of the Common Rule [Department of Health and Human Services (DHHS) 2001] to those studies, even though it has adopted the Common Rule for U.S. EPA–sponsored research (Silbergeld et al. 2004).

In February 2004, the NRC issued its report. It recommended that privately funded human dosing experiments for U.S. EPA regulatory purposes can be conducted only if they meet strict scientific and ethical standards and provide a public health or environmental benefit. It also recommended that the Common Rule should also apply to such research (NRC 2004). The NRC recommended that institutional review boards (IRBs) should review all proposed experiments to determine whether they meet appropriate scientific and ethical standards and that the U.S. EPA should establish a special review board to oversee these types of experiments. The NRC also stated that the U.S. EPA should not accept data from previous experiments, which it said did not meet scientific and ethical standards (NRC 2004).

On 3 November 2004, the U.S. EPA released a draft of a proposed plan for human testing. In the proposed plan, the U.S. EPA announced that it would evaluate data from industry-sponsored studies on a case-by-case basis “applying statutory requirements, the Common Rule, and high ethical standards as a guide, until such time as this practice is replaced by a rulemaking” (U.S. EPA 2004a, p. 6664). As soon as the U.S. EPA made this announcement, some commentators faulted the proposed plan for lack of consistency and enforceability (Associated Press 2004). However, the plan has helped clarify the U.S. EPA’s position on human testing by signaling its commitment to adhering to the Common Rule for all human experiments. The U.S. EPA plans to issue guidance for third-party researchers for adherence to the Common Rule and develop a final rule by 2006.

A variety of laws, including the Federal Food, Drug and Cosmetic Act (1999), the Federal Insecticide, Fungicide and Rodenticide Act (1964), and the Toxic Substances Control Act (1999) grant the U.S. EPA authority to regulate human exposures to environmental toxins in the United States, including pesticide residues on foods and in food additives. The U.S. EPA establishes safety levels for exposure to pesticides through a process known as pesticide registration (U.S. EPA 2004b). Before a manufacturer can sell a pesticide, it must register it with the U.S. EPA. In registering a pesticide, the U.S. EPA determines allowable human exposures of the pesticide, based on data submitted by pesticide manufacturers and federal agencies, as well as its own research. In arriving at an acceptable exposure, the U.S. EPA considers exposures from different sources, such as agricultural work and ingestion of food with traces of pesticides, as well as cumulative exposures (NRC 2004). Users of the pesticide, such as farmers and applicators, are required to comply with the U.S. EPA’s requirements for allowable human exposures.

The Food Quality Protection Act (FQPA), which President Clinton signed in 1996, amended existing laws pertaining to the U.S. EPA. Before the FQPA, the U.S. EPA regulated allowable pesticide exposure in food based on the no observable adverse effect level (NOAEL) in animal studies. After establishing a NOAEL in animals (usually rodents), the U.S. EPA would usually add a 10-fold interspecies safety factor to allow for differences between animals and humans, and a 10-fold intraspecies safety factor to account for variation in sensitivities among humans. Thus, the allowable exposure in human beings would usually be no more than 1% of the NOAEL exposure. The FQPA mandated an additional 10-fold increase in safety to account for variations between adults and children when there are no data to support a smaller safety factor. Therefore, under the FQPA, many chemicals would have an allowable exposure of no more than 0.1% of the NOAEL in animals. This change in the allowable exposure would have a significant impact not only on pesticide companies but also on agriculture, which depends heavily on pesticides. In implementing the law, the U.S. EPA has focused on 40 different organophosphates, which have been used to kill insects for many years.

Faced with higher safety standards for a variety of chemicals, some pesticide companies decided to conduct experiments on human subjects to produce data that they hoped would convince the U.S. EPA to lower the interspecies safety factor. From 1996 to 2004, the U.S. EPA received 20 studies from private companies providing human dosing data on pesticide toxicity (U.S. EPA 2004a). Thus, a law that was intended to provide additional safety protection for children had the unintended effect of encouraging some companies to test toxic compounds on human beings to avoid the regulatory impact of the law.

In the public debate surrounding pesticide testing on human subjects, two distinct positions have crystallized (Robertson and Gorovitz 2000). The first position, adopted by the NRC and others (NRC 2004; Oleskey et al. 2004), holds that pesticide testing on human subjects can be conducted, but only under the most stringent scientific and ethical standards, such as favorable benefit–risk ratios, informed consent, equitable subject selection, risk minimization, valid study design, and scientific necessity. The second position, adopted by environmental and public health interest groups, maintains that these experiments should be prohibited (Children’s Environmental Health Network 1999; EWG 1998; Sharav 2003).

In this commentary, we evaluate what we consider to be the strongest argument for prohibiting any testing of pesticides on human subjects—namely, that the benefits of the experiments are not significant enough to justify the risks posed to healthy subjects. We challenge this argument by exploring the benefits of pesticide testing for human health, discussing the scientific necessity of some experiments, and proposing ways to reduce the risks to subjects. We are not commenting on the studies that have been conducted. We accept Lockwood’s (2004) analysis that at least six of the human dosing studies submitted to the U.S. EPA were scientifically and ethically flawed. We are concerned here with the broader question of whether any type of experiment that intentionally exposes human subjects to pesticides can meet scientific and ethical standards.

## Benefits versus Risks in Research

One of the most important principles of ethical research is that the risks to the subjects must be justified by virtue of the benefits to the subject and to society (Emanuel et al. 2000; Levine 1988; Nuremberg Code 1949; World Medical Association 2000). The Common Rule codifies this principle: “Risks to subjects are reasonable in relation to anticipated benefits, if any, to subjects, and the importance of the knowledge that may reasonably be expected to result” [Common Rule (DHHS 2001)]. If the benefits of testing pesticides on human subjects do not outweigh the risks, then these experiments should not be conducted.

To determine whether the benefits of an experiment outweigh its risks, one must consider both sides of the benefit–risk ratio. In the experiments we are considering here, the subjects would be healthy individuals who would not stand to benefit medically or psychologically from participation. They may benefit economically from participation, but most agencies and commentators hold that it is not ethically appropriate to consider a financial incentive to participate in an experiment as a potential benefit in calculating the benefit–risk ratio [Food and Drug Administration (FDA) 1998; NIH 2004]. Because the subjects do not stand to benefit from the experiments, the benefits of these experiments hinge on their potential benefits to society, which are based on the value of the knowledge produced.

## Social Value

The principle that human experiments should have some redeeming social value has been an essential principle in human experimentation since the adoption of the Nuremberg Code (1949). Opponents of the pesticide experiments have argued that these experiments do not have any significant benefits for society. According to the EWG (1998, p. 13), “the degree to which society as a whole benefits from the use of specific pesticides, and pesticides generally, is the subject of heated debate. It is not obvious that these debatable social benefits alone would justify experimental risks to humans.” Richard Wiles, vice president for research for the EWG, also challenges the social benefits of the research: “This is not research designed to find a cure for a disease or to generate a new scientific advance” (Kamenetsky 2003, p. 1).

Even though the disputed experiments would not be designed to diagnose, treat, or prevent a disease, they could yield knowledge about the toxic effects of pesticides on humans, which could promote human health (NRC 2004). First, the knowledge obtained from the experiments could be used by the U.S. EPA to impose stricter safety standards on the chemicals under investigation. In some situations, a more than 10-fold interspecies safety factor may be required to protect the general human population or susceptible subpopulations (Cranor 1997). For this outcome to happen, it is important that the experiments have sufficient statistical power to demonstrate that a greater (or less) than 10-fold interspecies safety factor is needed for a particular chemical. Because pesticide companies, like drug companies, would have a strong financial motive for not reporting unfavorable results, steps should be taken to ensure that they do not suppress such findings (Angell 2004). All data from such studies submitted to the U.S. EPA should be publicly available within a reasonable time after completion of the studies.

Second, knowledge about how pesticides affect human beings can be useful in addressing human health issues outside of the U.S. EPA’s regulatory authority. People are exposed to pesticides in variety of different contexts, such as exposure from vehicles and clothing; exposure in public places that use pesticides; and exposure in the air, soil, and water. Knowledge about how pesticides affect human beings could be useful in taking measures to reduce pesticide exposure in areas that lie beyond the U.S. EPA’s domain and could encourage Congress to adopt new legislation to protect the public from pesticides.

Third, the proposed experiments may contribute to our understanding of the usefulness of animal models in toxicology testing because they would allow researchers to compare human and animal data. In toxicology research, scientists draw conclusions about the impacts of chemical on human health based on experiments in animals. For example, chemicals may be classified as carcinogens if they cause cancer in laboratory animals. Although animal models play an essential role in all toxicology testing, they do have some limitations due to differences in genetics, anatomy, and physiology between humans and different animal species (Brent 2004; Swanson et al. 2004). Understanding limitations of animal models may contribute to human health by improving our knowledge of the toxic effects of chemicals in human beings and contributing to effective regulation of pesticides, pharmaceuticals, and other compounds.

A critic of the studies might admit that there are some potential benefits from testing pesticides on human subjects, yet still maintain that the benefits are not great enough. One might argue that the benefits must be at least as great as the potential benefits of research that exposes healthy subjects to an equivalent amount of risk, such as Phase I clinical trials of new pharmaceuticals. We address this objection more fully below, when we evaluate the risks of human pesticide testing. At this juncture, however, we would like to point out that new drugs are not always beneficial, and that some cause a greater deal of harm, as demonstrated by Merck’s decision to withdraw Vioxx from the market (Miller 2005). In deciding whether to approve a new drug, the Food and Drug Administration weighs benefits and risks of the drug. If the risks are high, then the benefits must also be high. If the risks are low, then the benefits do not have to be as high. We argue below that the risks of some types of pesticide experiments, if implemented and monitored properly, can be low enough to justify the use of human subjects.

## Scientific Necessity

If the knowledge produced by pesticide experiments has some social value, the benefits of the experiments will not outweigh the risks if the knowledge can be obtained by other means. One of the key principles of research ethics is that human beings should not be used in experiments if those experiments are not scientifically necessary (Emanuel et al. 2000; Nuremberg Code 1949). If an experiment is not scientifically necessary, then the risks of the experiment outweigh the benefits of the experiment (Levine 1988). Critics of pesticide testing on human subjects hold that there is no need to conduct these experiments because scientists can obtain adequate data from experiments on animals, as well as studies on human beings that do not involve controlled experiments, such as epidemiologic or field studies (EWG 1998).

Without a doubt, epidemiologic studies and field studies can provide useful information about the effects of pesticides on human health. For example, an epidemiologic study by Kato et al. (2004) examined 376 cases and 463 controls from a cancer registry to determine whether pesticide exposure increases the risk of non-Hodgkin lymphoma (NHL) in women. The study found that women who worked on a farm where pesticides were used for at least 10 years had twice the risk of NHL in relation to a comparable group of women who did have this pesticide exposure. A similar epidemiologic study conducted by McDuffie et al. (2001) examined 517 cases and 1,506 controls of Canadian men from a variety of occupations. The study concluded that NHL is associated with several different pesticides. A field study conducted by Aprea et al. (1997) measured pesticides in the urine of agricultural workers 1, 5, and 11 days after exposure to pesticides during vine spraying and leaf thinning. The study compared the agricultural workers to a control group of 46 people who did not have the same exposure. Aprea et al. (1997) found that pesticide excretion was positively correlated with pesticide exposure, with the peak pesticide excretion the night after exposure. Coronado et al. (2004) performed a similar type of study, using a random sample of agricultural workers and their children. They measured pesticide residues and pesticide excretion in urine.

Although these studies and others like them provide scientists, clinicians, public health practitioners, and regulators with important knowledge, they have some limitations. First, they have many different uncontrolled variables that can confound data analysis and interpretation. In all of these studies, subjects were exposed to more than one type of pesticide as well as to many other types of potentially toxic chemicals. Exposures also were not uniform. The subjects had variations in diet, tobacco use, environmental temperature, water intake, alcohol use, and other factors that can affect health. Although epidemiologic and field studies can establish patterns and correlations, they cannot adequately prove causation. Kato et al. (2004) were careful to point out that their study showed the pesticides increase the risk of NHL but do not cause the disease. The randomized, controlled clinical trial is the gold standard for proving causation in clinical research (Sackett et al. 1997). Controlled trials also offer the best data concerning the effects of pesticides in humans.

Second, to conduct epidemiologic or field studies of pesticides, the products must already be on the market because one cannot measure natural exposures to a chemical that people are not using. Thus, epidemiologic and field studies do not provide regulators or clinicians with any information about a pesticide before its introduction. It would often be important to have better information about a pesticide before human populations are exposed to that pesticide, because this information could help promote human health and safety. Although the U.S. EPA examines animal data before making decisions about new compounds, the agency could also benefit from having access to human data.

The NRC (2004) recommended that three types of experiments on human beings could provide information not obtainable by other methods or means: *a*) pharmacokinetic (PK) studies, which are designed to elucidate how pesticides are absorbed, metabolized, and eliminated by the human body; *b*) pharmacodynamic (PD) studies, which are designed to elucidate how pesticides affect human physiology via their action on biomarkers; and *c*) studies that examine the psychological and behavioral effects of pesticides, such as nausea, dizziness, fatigue, or headache. According to the NRC (2004), the first two types of studies could be conducted at very low doses that would pose very low risks to subjects. The third type of study poses risks to human subjects, which can be minimized through proper population selection and protocol design, according to the NRC (2004).

We disagree somewhat with the NRC on these issues. For all these types of studies, it is possible to develop field studies, like the one conducted by Aprea et al. (1997), that are ethically less troubling than an intentional dosing study. One can take advantage of the fact that people expose themselves to pesticides to design experiments that measure the effects of pesticides on human beings. For example, carefully assessing blood concentrations before field entry by agricultural workers, followed by multiple time-point blood concentrations on leaving the field, could be used to determine overall absorption and elimination kinetics. Matching data from this type of study with PD measurements could eliminate the need for a clinical study that intentionally exposes individuals to pesticides. Although this type of study has many of the methodologic difficulties associated with classical epidemiology studies, such as confounding variables and bias, and some additional medical concerns, such as conducting the research in the field rather than in a clinical setting, it creates less of an ethical problem than an intentional dosing study because the subjects are already exposed to pesticides in their daily lives. These studies would pose few additional risks to subjects beyond those that they would already face in their environment.

Using field studies to obtain pesticide data has an important limitation, however: They do not provide information about pesticides that are not being used at all or that are not being used frequently enough to obtain reliable data. For the method to work, one must be able to recruit enough subjects to obtain reliable and statistically significant data. If one wants to obtain human data on a pesticide that is not being used at all or that is being used infrequently, one must intentionally expose human subjects to the chemical. Thus, we believe there are good reasons to conduct studies on pesticides that have not been introduced to the market or are not being used frequently enough to obtain reliable data from field studies. Only these types of intentional dosing studies are scientifically necessary.

## Risk and Safety

If the experiments have social value and are scientifically necessary, they will still not be ethical unless the risks are low enough to yield a favorable benefit–risk ratio. The benefits of the experiments, though significant, are probably not as high as the benefits of a clinical study on a new medical therapy. Could the risks be kept low enough that the benefits would outweigh them? To address this question, it is important to understand the dosing regimen of the studies and compare it to the dosing regimen used in Phase I trials on healthy subjects. We realize that the comparison to Phase I drug trials is not completely apt, because pesticides will not be used to diagnose, treat, or prevent human diseases. However, we make the comparison as a way of understanding aspects of the studies related to toxic chemical exposures.

A Phase I study occurs after extensive animal testing to determine whether the drug is safe enough to test on human subjects. The goal of a Phase I trial of a new drug is to determine its safety for human use. Phase I studies usually are conducted on healthy volunteers, although some Phase I studies are conducted on very ill subjects, such as patients with advanced cancer. Phase I studies follow a dose-escalation regimen designed to determine the maximum tolerable dose (MTD). The MTD for a particular subject is the dose at which the drug causes toxicity or at which the subject experiences intolerable symptoms, such as nausea, pain, or difficulty breathing. The pesticide experiments that we have been discussing would be designed not to measure the MTD for a chemical, but to measure the NOAEL (i.e., the level of exposure to the chemical at which the subject has no observable adverse effects). To measure the NOAEL, the experiments escalate the exposure level until some predefined effect is observed, such as an effect on a biomarker, specific levels of the chemical in the subject’s blood or urine, or symptoms such as nausea, dizziness, or headache. The adverse effects could be measured by giving the subjects very low exposures and then stopping the escalation as soon an adverse effect is observed.

Would these types of experiments be safe enough? The NRC (2004) concluded that studies to measure NOAELs for pesticides would probably be at least as safe as studies designed to measure MTDs for drugs. One might argue that short-term risks of exposing people to low levels of pesticide would be lower than the risks of exposing people to toxic levels of drugs, since an observable adverse effect is safer than toxicity. But what about the long-term effects of pesticide experiments? Unfortunately, we are not aware of any data on the long-term risks of intentionally exposing human subjects to low doses of pesticides for a short period of time. However, data from other types of studies indicate that there could be some significant long-term risks of limited exposures to pesticides because pesticides can induce mutations that cause cancer and may have adverse impacts on the neuromuscular, cardiovascular, and endocrine systems (NRC 2004). To minimize long-term risks from intentionally exposing human subjects to pesticides, we recommend that human subjects should not be exposed to pesticides that are known carcinogens or that are known to cause permanent damage to human tissues or organs in low doses.

We agree with the NRC (2004) that pesticide experiments on human subjects should not be conducted if the pesticides are expected to cause serious or irreversible harm to human subjects. The experiments can be conducted only if the harms they are expected to produce are not serious and are reversible. For example, the presence of a pesticide in the blood or urine is an effect that is not serious and is reversible because the body will continue to eliminate the pesticide. Tissue or organ damage, however, might be serious or irreversible. We also think that the burden of proof should be on the researchers to prove that a proposed study is not expected to produce effects that are serious or irreversible. IRBs should assume that intentionally exposing human subjects to even small doses of pesticides may produce serious or irreversible effects, unless the researchers produce evidence to the contrary.

To minimize all of the risks from the experiments discussed herein, we recommend the following safety measures, most of which have also been endorsed by the NRC (2004):

1. The experiments should take place in a clinical setting, supervised by medical personnel.
2. Subjects should be carefully selected and monitored.
3. The studies should exclude subjects who are pregnant, are unhealthy, or have significant pesticide exposures in their daily lives.
4. Extensive animal testing should take place to determine exposure levels that are not likely to cause any serious or permanent damage to subjects.
5. Escalation of exposure levels should proceed cautiously and stop as soon as a well-defined, observable adverse effect is detected or as soon as the expected maximum human exposure in food, water, or the environment is achieved.
6. Independent data and safety monitoring boards (DSMBs) should be established to monitor risks to subjects and protect them from harm.
7. Researchers should have a clear definition of an “adverse event” and immediately report adverse events to the IRB, the DSMB, research sponsors, and the U.S. EPA.
8. Subjects should be fully informed of the risks of participation.

## Conclusion

The strongest argument against any pesticide testing on human subjects is that the benefits of the research do not outweigh the risks. [In our supplemental material (http://ehp.niehs.nih.gov/members/2005/7720/suppl.pdf), we evaluate three other arguments against testing pesticides on human subjects.] In this article, we have attempted to rebut this argument by showing that in some types of studies, the benefits would outweigh the risks. Such studies must meet at least four stringent conditions [the supplemental material (http://ehp.niehs.nih.gov/members/2005/7720/suppl.pdf) contains a more complete list]:

1. The knowledge gained from the study is expected to promote human health.
2. The knowledge cannot be reasonably obtained by other means.
3. The study is not expected to cause serious or irreversible harm to the subjects.
4. Appropriate safeguards are in place to minimize harm to the subjects.

Because we think that some of the experiments discussed in this article could meet these conditions, we do not support a ban on experiments that intentionally expose human subjects to pesticides, and we support the U.S. EPA’s decision to move forward with rule making and guidance in this area.
